# Synergism interaction between genetic polymorphisms in drug metabolizing enzymes and NSAIDs on upper gastrointestinal haemorrhage: a multicenter case-control study

**DOI:** 10.1080/07853890.2021.2016940

**Published:** 2022-02-04

**Authors:** Narmeen Mallah, Maruxa Zapata-Cachafeiro, Carmelo Aguirre, Eguzkiñe Ibarra-García, Itziar Palacios-Zabalza, Fernando Macías-García, María Piñeiro-Lamas, Luisa Ibáñez, Xavier Vidal, Lourdes Vendrell, Luis Martin-Arias, María Sáinz-Gil, Verónica Velasco-González, Manuel Bacariza-Cortiñas, Angel Salgado, Ana Estany-Gestal, Adolfo Figueiras

**Affiliations:** a Department of Preventive Medicine, University of Santiago de Compostela, Santiago de Compostela, Spain; b WHO Collaborating Centre for Vaccine Safety, Santiago de Compostela, Spain; c Genetics, Vaccines and Pediatric Infectious Diseases Research Group (GENVIP), Instituto de Investigación Sanitaria de Santiago de Compostela, Santiago de Compostela, Spain; d Centro de Investigación Biomédica en Red de Enfermedades Respiratorias (CIBER-ES), Carlos III Health Institute, Madrid, Spain; e Consortium for Biomedical Research in Epidemiology and Public Health (CIBER en Epidemiología y Salud Pública-CIBERESP), Carlos III Health Institute, Madrid, Spain; f Pharmacotherapy Group, Biocruces Bizkaia Health Research Institute, Barakaldo, Spain; g Basque Country Pharmacovigilance Unit, University Hospital of Galdakao-Usansolo, Osakidetza, Spain; h Pharmacology Department, Medicine and Nursing Faculty, University of the Basque Country, Barakaldo, Spain; i Osakidetza Basque Health Service, Pharmacy Department, Urduliz Hospital, Urduliz, Spain; j Department of Gastroenterology and Hepatology, University Hospital of Santiago de Compostela, Santiago de Compostela, Spain; k Health Research Institute of Santiago de Compostela (IDIS), Santiago de Compostela, Spain; l Department of Pharmacology, Therapeutics and Toxicology, Catalonian Institute of Pharmacology, Clinical Pharmacology Service, Vall d’Hebron University Teaching Hospital, Autonomous University, Barcelona, Spain; m Centre for Research on Drug Safety (CESME), Valladolid University, Valladolid, Spain; n Centro de Saúde de Vite, Santiago de Compostela, Spain

**Keywords:** Aspirin, genetic variation, interaction, non-steroidal anti-inflammatory drugs, upper gastrointestinal haemorrhage

## Abstract

**Background:**

Interindividual genetic variations contribute to differences in patients’ response to drugs as well as to the development of certain disorders. Patients who use non-steroidal anti-inflammatory drugs (NSAIDs) may develop serious gastrointestinal disorders, mainly upper gastrointestinal haemorrhage (UGIH). Studies about the interaction between NSAIDs and genetic variations on the risk of UGIH are scarce. Therefore, we investigated the effect of 16 single nucleotide polymorphisms (SNPs) involved in drug metabolism on the risk of NSAIDs-induced UGIH.

**Materials and methods:**

We conducted a multicenter case-control study of 326 cases and 748 controls. Participants were sub-grouped into four categories according to NSAID exposure and genetic profile. We estimated odds ratios (ORs) and their 95% confidence intervals (CI) using generalized linear mixed models for dependent binomial variables and then calculated the measures of interaction, synergism index (S), and relative excess risk due to interaction (RERI). We undertook stratified analyses by the type of NSAID (aspirin, non-aspirin).

**Results:**

We observed an excess risk of UGIH due to an interaction between any NSAID, non-aspirin NSAIDs or aspirin and carrying certain SNPs. The greatest excess risk was observed for carriers of: rs2180314:C>G [any NSAID: *S* = 3.30 (95%CI: 1.24–8.80), RERI = 4.39 (95%CI: 0.70–8.07); non-aspirin NSAIDs: *S* = 3.42 (95%CI: 1.12–10.47), RERI = 3.97 (95%CI: 0.44–7.50)], and rs4809957:A>G [any NSAID: *S* = 2.11 (95%CI: 0.90–4.97), RERI = 3.46 (95%CI: −0.40–7.31)]. Aspirin use by carriers of rs6664:C>T is also associated with increased risk of UGIH [OR_aspirin(+),wild-type_: 2.22 (95%CI: 0.69–7.17) *vs.* OR_aspirin(+),genetic-variation_: 7.72 (95%CI: 2.75–21.68)], yet larger sample size is needed to confirm this observation.

**Conclusions:**

The joint effect of the SNPs s2180314:C>G and rs4809957:A>G and NSAIDs are more than three times higher than the sum of their individual effects. Personalized prescriptions based on genotyping would permit a better weighing of risks and benefits from NSAID consumption.

**KEY MESSAGES**
- Multicenter case-control study of the effect of genetic variations involved in drug metabolism on upper gastrointestinal haemorrhage (UGIH) induced by NSAIDs (aspirin and non-aspirin).
- There is a statistically significant additive synergism interaction between certain genetic polymorphisms and NSAIDs on UGIH: rs2180314:C>G and rs4809957:A>G. The joint effect of each of these single nucleotide polymorphisms and NSAIDs on UGIH is more than three times higher than the sum of their individual effects.
- Genetic profiling and personalized prescriptions would be useful in managing the risks and benefits associated with NSAIDs.

## 1. Introduction

Adverse effects of non-steroidal anti-inflammatory drugs (NSAIDs) were associated with heavy health and economic burdens [1,2]. Upper gastrointestinal haemorrhage (UGIH) is a frequent adverse effect of NSAID treatment that can be life-threatening [3]. Nonetheless, NSAIDs continue to be the most prescribed drugs worldwide [4].

Furthermore, it is well-established that aspirin plays an important prophylactic role against highly incident diseases that are associated with elevated mortality rates, such as several types of cancer and cardiovascular events [5–12]. Nevertheless, the association of aspirin with gastrointestinal bleeding has discouraged the adoption of this drug as a general prophylactic measure against disorders with great public health impact [13]. In addition, gastrointestinal symptoms in patients who used aspirin to protect against cardiovascular events had led to treatment interruption [14], and consequently to an increase in cardiovascular risk [15,16].

Marked interindividual differences with respect to their response to NSAIDs have long been recognized and attributed to many factors including genetic variations in metabolizing enzymes [17–20]. Several studies also reported a possible relationship between genetic variations in users of NSAIDs and gastrointestinal disorders [21–27]. In this context, genetic pharmacokinetic factors are of special importance since variations in genes involved in drug metabolism might alter their expression and thus increase the risk of undesirable effects like bleeding and cardiovascular events. Therefore, identifying patients at risk of UGIH based on their genetic background and personalized NSAID prescriptions might help weigh the risks and benefits associated with each type of NSAIDs and thus avoid adverse effects in susceptible individuals.

Currently, there is a lack of knowledge about the effect of variations in genes involved in drug metabolism on the risk of UGIH in general, and in NSAID users in specific. Taking into account the considerable morbidity and mortality rates of UGIH [28,29], and the wide spectrum of NSAID benefits, we carried out a multicenter case-control study that primarily aimed at testing the modification effect of 16 genetic polymorphisms involved in drug metabolism on the risk of NSAIDs-related UGIH. As a secondary objective of this study, we investigated the modification effect of those 16 genetic polymorphisms on the risk of non-aspirin NSAIDs-related UGIH as well as on aspirin-related UGIH.

## 2. Materials and methods

### 2.1. Study settings and design

This study represents a continuation of a previous full case-control study (i.e. case-control encompassing exposed and non-exposed patients to NSAIDs, on the contrary to other partial case-control studies that include only exposed patients) [30], published elsewhere, and shares the same protocol [31,32]. Patients were recruited from four hospitals in Spain (Barcelona, Galdakao, Santiago de Compostela, and Valladolid), between January 2004 and November 2007 and between January 2013 and October 2015. The study protocol was approved by the ethics committee of each participating centre (Barcelona: CEIC protocol number: Es38121226Z; Euskadi: CEIC-E protocol number: PI2013101; Galicia: CEIC-G protocol number: 2013/263 and Valladolid: CEIC-VA-ESTE-HCUV protocol number: PI-14-142). The participants provided written informed consent before enrolment in the study.

### 2.2. Definition of cases and controls

Cases were patients admitted to the hospital with symptoms of UGIH that were diagnosed surgically or endoscopically. Eligible cases were included irrespective of the grade of UGIH severity.

For each case, controls matched by the hospital, gender, and age (±5 years) were selected. To avoid selection bias due to excessive intake of NSAIDs, controls were either outpatients or patients enrolled from the preoperative unit among subjects who were about to undergo any of the following non-painful mild surgeries which were unrelated to the use of NSAIDs: plastic surgery, inguinal or umbilical hernia (strangulated or programmed), lipoma, varicotomy, prostatic adenoma, prostatic hyperplasia, thyroid nodules and thyroglossal cyst (euthyroid), eye cataract, phimosis, ear pinning, vocal cord cyst, tubal ligation, and septoplasty.

To ensure that all subjects belong to the same source of population, they were recruited from patients and outpatients attended by the same hospitals. All patients were biologically unrelated. The analysis was restricted to European participants to control for the risk of stratification bias [33]. We used the native language of the participants and their parents as a proxy of ethnicity [34–37]. Patients with a history of neoplasia, liver cirrhosis, or coagulopathy were excluded to control for the risk of Berkson’s bias [38]. The inclusion and exclusion criteria of the cases and controls are specified in more detail in Table 1.

**Table 1.** Motives of the exclusion of cases and controls from the study.

Reasons of exclusion^†^	EMPHOGEN I (2004–2007)	EMPHOGEN II (2013–2015)
CASES (*N* = 3731)	**3120**	**611**
Primary exclusions (*N* = 2655)	**2147**	**508**
Age < 18	31	2
Excludable endoscopic diagnosis^‡^	1213	377
History of UGIH	121	18
Intrahospital UGIH	89	5
UGIH without endoscopic or surgical diagnosis from admission to discharge	121	3
Nasogastric or percutaneous tube carrier	75	2
<3 months’ residence in study area	42	7
Admission time <24 h	208	8
Admission not due to UGIH	154	80
Death	0	2
Other	93	4
Secondary exclusions (*N* = 744)	**646**	**98**
Refusal to sign informed consent form	21	0
Occurred at weekend or vacations period	57	21
Death	11	2
Endoscopy performed more than 48 h after admission	83	39
Discharge from hospital or visit to healthcare facility in the 15 days prior to admission	54	20
Severe condition	7	1
Psychological disorders	12	4
Illiterate	2	0
Deaf or blind	1	0
Lives in a residence or closed institution and does not know the drugs taken	7	1
Refusal to answer or failure to complete the interview	12	5
Impossible to conduct interview within the 15-day period preceding admission	6	4
Admission time <24 h	0	1
Other	373	0
Excluded from analysis (*N* = 332)	**327**	**5**
Non-white patients	4	0
Unavailable biological material	323	5
CONTROLS (*N* = 1073)	**1071**	**2**
Refused to sign informed consent form	45	0
Age < 18	1	0
History of disease	11	1
Intrahospital UGIH	89	0
Nasogastric or percutaneous tube carrier	2	0
<3 months' residence in study area	1	0
Severe condition	1	0
Psychological disorders	1	0
Deaf or blind	3	0
Refusal to answer or failure to complete the interview	80	0
Impossible to conduct interview within the 15-day period preceding admission	60	0
Date of last admission	0	1
Other	13	0
Non-white patients	15	0
Unavailable biological material	749	0

^†^Cases and controls were excluded upon presenting one or more exclusion criteria.

^‡^Excludable endoscopic diagnosis included gastritis, esophagitis, oesophageal varices, gastric or duodenal neoplasia, Mallory-Weiss syndrome, angiodysplasia, anastomotic ulcers, diverticulitis, acute alcohol intoxication, hiatal hernia, and papule.

Bold values represent the total per reason of exclusion group and study period.

### 2.3. Data collection

Both cases and controls were thoroughly interviewed by trained health personnel, using a questionnaire specifically designed for this study. The collected data include participants’ sociodemographic characteristics, clinical antecedents, smoking habits, alcohol and caffeine consumption, the motive for hospital admission, underlying symptomatology (for cases), the motive for the scheduled surgery (for controls), previous episodes of gastric diseases, and exposure to pharmaceutical drugs (including the medicine’s daily dose and indication). Direct relatives or healthcare assistants, who took care of the patient’s medication, could attend and participate in the interview, but only data confirmed by the patient were considered. When the participant was not able to remember any of the requested information, the interview was repeated on a posterior date, or the patient was contacted by telephone if s/he had been discharged from the hospital. In case the patient doubted or was uncertain about specific information, that information was confirmed later by consulting the medical records of the patient.

Index dates were established to ascertain any exposure to NSAIDs. Information on NSAID exposure was extracted from patients’ medical records, but the researchers were blind to patients’ use of NSAIDs. For the cases, the index date was the day of onset of the first signs or symptoms of UGIH, while for the controls it was the day of the interview. NSAIDs exposure was considered if the consumption took place in the week preceding the index date [39–41]. For ease of recall, a catalog of prompt cards of the most consumed NSAID boxes was shown to the participants during the interview.

The reliability of the interview was rated on a scale of 0–10 as perceived by the interviewer, where zero means that the answers provided by the patient were completely unreliable. Patients whose interview was rated by zero were excluded from the study.

A 5 ml blood sample was withdrawn from each participant and stored in EDTA tubes or as spots on IsoCode papers at −80 °C until genotyping.

### 2.4. Risk factors associated with UGIH

The following co-variables which were known to affect the risk of UGIH were considered: (1) previous infection with *Helicobacter pylori*; (2) therapeutic groups, such as proton pump inhibitors or oral anticoagulants; (3) digestive system disorders classified according to the patient's history of ulcer and bleeding (none or dyspepsia; ulcer; or bleeding); and (4) the reliability of the interview.

### 2.5. Helicobacter pylori determination

The presence of anti-*H. pylori* IgG antibodies in human serum were determined using the commercial ELISA kits: Human Anti-Helicobacter pylori IgG ELISA Kit (ab108736, Abcam, Cambridge, England), and Captia™ *H. pylori* IgG EIA (ref: 2346400, Trinity Biotech Captia, Co. Wicklaw, Ireland), and following the manufacturer's protocol. The participants were inquired if they had previously been treated against *H. pylori* infection to avoid any false-positive results caused by old infections.

### 2.6. Single nucleotide polymorphisms (SNPs) selection and genotyping

A comprehensive list of SNPs involved in gastrointestinal disorders (bleeding or ulcer) was retrieved by reviewing research reports published in MEDLINE until April 2017. The reference numbers (rs number) of the selected SNPs were confirmed using PubMed [42]. Subsequently, the function of the corresponding genes and the clinical significance of the genetic variations were identified through a literature review. Finally, SNPs in genes that may influence drug metabolism were selected for genotyping [25,26].

DNA was extracted from blood stored in EDTA tubes using chemagic™ DNA Buffy Coat 200 Kit H96 (PerkinElmer, reference number CMG-713) and from blood spots using chemagic™ DNA Blood 200 Kit H96 (PerkinElmer, reference number CMG-717). Extracted DNA was then quantified using Quant-iT™ PicoGreen™ dsDNA Assay Kits (ThermoFisher Scientific, reference number P7589). DNA concentration was normalized at 10–20 ng/µl in a minimum total volume of 40 µl. Samples were genotyped in a phonotype-blind process. iPlex^®^ Gold chemistry and MassARRAY platform were used according to the manufacturer’s instructions (Agena Bioscience, San Diego, USA). Genotyping assays were designed using the Agena Bioscience MassARRAY Assay Designer 4.1 software. All assays were performed in 384-well plates, including negative controls and a trio of Coriell samples for quality control. The reproducibility of 7% of the samples was also checked between and/or within plates.

The compliance of the SNPs with Hardy–Weinberg equilibrium was checked using the SNPassoc Library of the R package (Version 1.9-2) [43–45]. In addition, all cluster plots were manually inspected by trained personnel using MassArray Typer software.

### 2.7. Statistical analysis

To determine any interaction between each of the 16 SNPs and NSAID exposure on the risk of UGIH, participants were grouped according to their genotype and NSAID exposure. Stratified analysis by the type of NSAID (any NSAID, non-aspirin NSAIDs, and aspirin) was carried out. In each analysis, the following four groups of participants were obtained: [group 1: drug(+), wild-type; group 2: drug(+), genetic-variation; group 3: drug(−), genetic-variation; and group 4: drug(−), wild-type]. Adjusted odds ratios (ORs) of UGIH were calculated in each group and then checked for any potential interaction between the presence of a genetic variation and drug exposure. The group of subjects who were not exposed to the studied drug category (any NSAID, non-aspirin NSAIDs, or aspirin) and who were carriers of the wild-type genotype of the analyzed SNP (group 4) was used as the reference category for the estimations of the interactions.

ORs and their 95% confidence intervals (CI) were estimated by generalized linear mixed models for dependent binomial variables [46]. In the construction of the models, patients were placed at level 1; the strata (each case and its matched controls) at level 2; the hospital at level 3; and the period of patients’ recruitment at level 4. A random-effects model was used to examine the effect of the patients’ recruitment period, and a nested random-effects model was applied for the strata of cases and controls and health centre. The lmer function of the lme4 R package (version 1.1-21) was applied in the estimation of the models [47]. Potential confounding variables were introduced in the model if they modified the OR of the main variable by at least 10% and provided that the Schwartz's Bayesian Information Criterion improved [48].

The recommendations given by Knol and colleagues were followed to explore any potential interaction between NSAIDs and genetic polymorphisms, whereby we estimated the relative excess risk due to interaction (RERI) and the synergism index (S) along with their 95% CI [49–52].

## 3. Results

### 3.1. Clinical data collection

**Figure 1.**
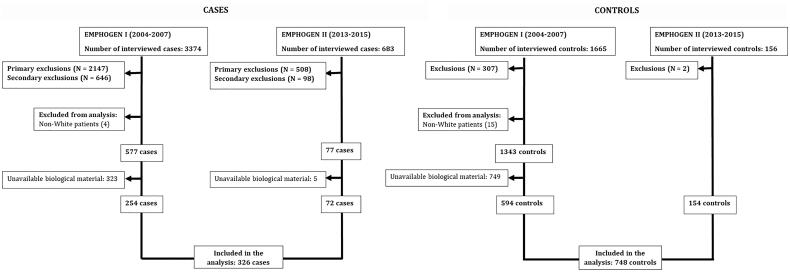
Flow of the cases and the controls throughout the two stages of the project.

One thousand and seventy-four patients (326 cases and 748 controls) fulfilled the inclusion criteria and were included in the final analysis. The flow of subjects and the motives of exclusion are presented in Figure 1 and Table 1. The patients’ demographic and clinical characteristics are presented in Table 2.

**Table 2.** Description of the cases and controls included in the study.

Characteristic	Cases (*N* = 326) № (%)	Controls (*N* = 748) № (%)	OR (95% CI)	*p*-Value
Age				
<45	41 (12.6%)	95 (12.7%)	1	
45–65	117 (35.9%)	271 (36.2%)	1.05 (0.68–1.63)	.8327
>65	161 (49.4%)	370 (49.5%)	1.01 (0.66–1.54)	.9757
missing	7 (2.1%)	12 (1.6%)		
BMI				
Underweight	10 (3.1%)	24 (3.2%)	0.74 (0.33–1.62)	.4588
Normal weight	114 (35.0%)	204 (37.3%)	1	
Overweight	128 (39.3%)	374 (50.0%)	0.61 (0.45–0.83)	.0201
Obese	68 (20.9%)	144 (19.3%)	0.85 (0.58–1.24)	.8676
Missing	6 (1.8%)	2 (0.3%)		
Gender				
Male	236 (72.4%)	559 (74.7%)	1	
Female	87 (26.7%)	189 (25.3%)	1.18 (0.87–1.6)	.2852
Missing	3 (0.9%)	0		
Arthrosis				
No	219 (67.2%)	469 (62.7%)	1	
Yes	86 (26.4%)	216 (28.9%)	0.86 (0.63–1.17)	.3303
Missing	21 (6.4%)	63 (8.4%)		
*Helicobacter pylori*				
No or uncertain	27 (8.3%)	138 (18.4%)	1	
Yes	276 (84.7%)	574 (76.7%)	2.54 (1.62–3.99)	<.0001
Missing	23 (7.1%)	36 (4.8%)		
Source of information				
Patients	259 (79.4%)	672 (89.8%)	1	
Healthcare assistant/direct relative	67 (20.6%)	76 (10.2%)	2.35 (1.62–3.42)	<.0001
Interview variables				
Number of interviews conducted				
1	274 (84.0%)	644 (86.1%)	1	
≥2	52 (16.0%)	104 (13.9%)	1.27 (0.81–1.99)	.2927
Reliability of the interview				
<5	13 (4.0%)	20 (2.7%)	1	
5–7	36 (11%)	78 (10.4%)	0.67 (0.29–1.54)	.3466
7–9	134 (41.1%)	310 (41.4%)	0.70 (0.33–1.48)	.3540
≥9	143 (43.9%)	340 (45.5%)	0.67 (0.31–1.41)	.2855
Personal history of gastrointestinal disorders				
None or dyspepsia	208 (63.8%)	647 (86.5%)	1	
Ulcer	48 (14.7%)	56 (7.5%)	2.74 (1.79–4.21)	<.0001
Bleeding	70 (21.5%)	45 (6.0%)	4.79 (3.15–7.27)	<.0001
Co-medications with drugs that are not NSAIDs				
Analgesics not narcotics				
No	272 (83.4%)	692 (92.5%)	1	
Yes	54 (16.6%)	56 (7.5%)	2.74 (1.81–4.15)	<.0001
Inhibitors of the proton pump				
No	290 (89.0%)	682 (91.2%)	1	
Yes	36 (11.0%)	66 (8.8%)	1.2 (0.77–1.88)	.4202
Antiaggregant				
No	261 (80.1%)	662 (88.5%)	1	
Yes	65 (19.9%)	86 (11.5%)	1.94 (1.34–2.8)	.0005
Anticoagulants				
No	291 (89.3%)	716 (95.7%)	1	
Yes	35 (10.7%)	32 (4.3%)	3.09 (1.85–5.15)	<.0001
Inhibitors of COX2				
No	323 (99.1%)	742 (99.2%)	1	
Yes	3 (0.9%)	6 (0.8%)	0.88 (0.21–3.77)	.8659

### 3.2. Genotyping

All genotyped samples were included in the analysis. The reproducibility of the 7% replicated random samples was 100%. All SNPs showed an acceptable genotype call rate: ≥98%. Both the calculations of the Hardy–Weinberg equilibrium (*p* < .001) and the manual inspection of the cluster plots confirmed that the controls were in equilibrium in terms of the corresponding polymorphisms (Table 3).

**Table 3.** Prevalence of the studied genotypes and Hardy–Weinberg equilibrium test.

Gene	Single nucleotide polymorphism reference number	Genotypes	Cases *N* (%)	Controls *N* (%)	Hardy–Weinberg equilibrium *p*-value
CYP4F11, cytochrome P450 family 4 subfamily F member 11	rs1060463	CC	64 (19.6)	127 (17.0)	0.03
		CT	165 (50.6)	398 (53.2)	
		TT	97 (29.8)	223 (29.8)	
CYP2A6, cytochrome P450 family 2 subfamily A member 6	rs28399433	AA	288 (88.3)	662 (88.5)	Not applicable
		AC	36 (11.0)	80 (10.7)	
CYP2B6, cytochrome P450 family 2 subfamily B member 6	rs36079186	TT	326 (100.0)	748 (100.0)	Not applicable
CYP4F11, cytochrome P450 family 4 subfamily F member 11	rs3765070	AA	65 (19.9)	128 (17.1)	0.03
		AG	165 (50.6)	398 (53.2)	
		GG	96 (29.4)	222 (29.7)	
CYP2A7, cytochrome P450 family 2 subfamily A member 7	rs3869579	AA	94 (28.8)	214 (28.6)	0.05
		AG	147 (45.1)	346 (46.3)	
		GG	85 (26.1)	188 (25.1)	
CYP11B2, cytochrome P450 family 11 subfamily B member 2	rs4536	CT	7 (2.1)	22 (2.9)	Not applicable
		TT	318 (97.5)	725 (96.9)	
CYP24A1, cytochrome P450 family 24 subfamily A member 1	rs4809957	AA	200 (61.3)	450 (60.2)	0.09
		AG	108 (33.1)	271 (36.2)	
		GG	18 (5.5)	27 (3.6)	
CYP2F1, cytochrome P450 family 2 subfamily F member 1	rs58285195	CC	2 (0.6)	1 (0.1)	0.62
		CT	29 (8.9)	51 (6.8)	
		TT	295 (90.5)	696 (93.0)	
GSTP1, glutathione S-transferase pi 1	rs1695	AA	132 (40.5)	321 (42.9)	0.52
		AG	157 (48.2)	332 (44.4)	
		GG	37 (11.3)	95 (12.7)	
GSTA2, glutathione S-transferase alpha 2	rs2180314	CC	50 (15.3)	104 (13.9)	0.13
		CG	139 (42.6)	318 (42.5)	
		GG	129 (39.6)	309 (41.3)	
GSTA1, glutathione S-transferase alpha 1	rs4715332	AA	104 (31.9)	259 (34.6)	0.29
		AC	159 (48.8)	374 (50.0)	
		CC	63 (19.3)	114 (15.2)	
GSTA5, glutathione S-transferase alpha 5	rs4715354	AA	55 (16.9)	143 (19.1)	0.71
		AG	160 (49.1)	362 (48.4)	
		GG	110 (33.7)	243 (32.5)	
NAT2, N-acetyltransferase 1	rs1799931	AA	1 (0.3)	1 (0.1)	0.45
		AG	16 (4.9)	40 (5.3)	
		GG	309 (94.8)	707 (94.5)	
CHST2, carbohydrate sulfotransferase 2	rs6664	CC	177 (54.3)	419 (56.0)	0.34
		CT	128 (39.3)	275 (36.8)	
		TT	21 (6.4)	54 (7.2)	
ALB_c, albumin	rs3756067	AA	37 (11.3)	91 (12.2)	0.33
		AG	136 (41.7)	320 (42.8)	
		GG	145 (44.5)	332 (44.4)	
SLCO3A1, solute carrier organic anion transporter family member 3A1	rs2283458	AA	33 (10.1)	100 (13.4)	1.00
		AG	169 (51.8)	348 (46.5)	
		GG	124 (38.0)	300 (40.1)	

### 3.3. Risk estimation and modification of effect

The odds of UGIH varied according to the genotype and NASID (aspirin or non-apsirin) exposure.

#### 3.3.1. Genotypes associated with high excess of risk of UGIH

The presence of certain genetic variations increases the odds of UGIH in users of any NSAID, non-aspirin NSAIDs, or aspirin as compared to users with wild-type genotypes (Table 4).

***rs2180314:C>G:*** Any NSAID use by carriers of rs2180314:C>G is associated with substantially higher odds of UGIH in comparison with NSAID users carrying the wild-type genotype [OR_drug(+),wild-type_: 3.17 (95%CI: 1.79–5.63) *vs.* OR_drug(+),genetic variation_: 7.30 (95%CI: 4.27–12.48)]. The measures of interaction showed a statistically significant high excess risk of UGIH from the interaction between NSAID and rs2180314:C>G [*S* = 3.30 (95%CI: 1.24–8.80), RERI = 4.39 (95%CI: 0.70–8.07)]. Similar findings were observed when the analysis was stratified by the type of NSAID: non-aspirin NSAIDs [*S* = 3.42 (95%CI: 1.12–10.47), RERI = 3.97 (95%CI: 0.44, 7.50)] and aspirin [*S* = 7.65 (95%CI: 0.81, 72.33), RERI = 8.39 (95%CI: −4.20, 20.99)], though the interaction estimates did not reach statistical significance in aspirin category probably due to the limited number of aspirin users.

***rs4809957:A>G:*** Substantially higher ORs of UGIH were observed for patients carrying rs4809957:A>G who are on treatment involving any NSAID [OR_wild-type:_ 4.12 (95%CI: 2.18–7.79) *vs.* OR_genetic-variation_: 7.57 (95%CI: 4.43–12.93)], or non-aspirin NSAID [OR_wild-type:_ 3.99 (95%CI: 2.06–7.75) *vs.* OR_genetic-variation_: 7.15 (95%CI: 4.10–12.46] in comparison with drug users carriers of the wild type genotype (Table 4). This excess in risk is suggested by the interaction estimates which are on the borderline of statistical significance: any NSAID [*S* = 2.11 (95%CI: 0.9–4.97); RERI = 3.46 (95%CI: −0.40–7.31)], non-aspirin NSAIDs [*S* = 2.03 (95%CI: 0.81–5.08); RERI = 3.11 (95%CI: −0.82–7.05)]. The interaction estimates for aspirin exposure—rs4809957:A>G are inconclusive due to the limited number of observations (Table 4).

**Table 4.** Odds ratios (OR) for UGIH stratified by patients’ genotype and NSAID (any NSAID, aspirin, non-aspirin) exposure and their interaction represented by synergism index (S) and relative excess risk due to interaction (RERI).

SNP (reference number)	Wildtype genotype		Genetic variation		RERI (95% CI)	S (95% CI)
	*N* (%) (cases/controls)	OR^†^ (95% CI); *p*-value	*N* (%) (cases/controls)	OR^†^ (95% CI); *p*-value		
rs2180314:C > G						
Any NSAID (No)	91 (26.1)/258 (73.9)	1	99 (21.4)/363 (78.6)	0.74 (0.50, 1.09); *p* = .1229	4.39 (0.70, 8.07)	3.3 (1.24, 8.8)
Any NSAID (Yes)	48 (44.4)/60 (55.6)	3.17 (1.79, 5.63); *p* = .0001	80 (61.5)/50 (38.5)	7.30 (4.27, 12.48); *p* < .0001		
Non-aspirin NSAID (No)	100 (27.2)/267 (72.8)	1	110 (22.9)/370 (77.1)	0.75 (0.52, 1.09); *p* = .1343	3.97 (0.44, 7.50)	3.42 (1.12, 10.47)
Non-aspirin NSAID (Yes)	39 (43.3)/51 (56.7)	2.89 (1.57, 5.31); *p* = .0006	69 (61.6)/43 (38.4)	6.61 (3.82, 11.46); *p* < .0001		
Aspirin intake (No)	115 (30.4)/263 (69.6)	1.0	142 (28.4)/358 (71.6)	0.92 (0.66–1.30); *p* = .6481	8.39 (−4.20, 20.99)	7.65 (0.81–72.33)
Aspirin intake (Yes)	10 (47.6)/11(52.4)	2.34 (0.84–6.49); *p* = .1031	14 (70.0)/6 (30.0)	10.65 (3.27–34.71); *p* = .0001		
rs4809957:A > G						
Any NSAID (No)	68 (23.4)/222 (76.6)	1	127 (23.5)/414 (76.5)	0.99 (0.66, 1.47); *p* = .9515	3.46 (−0.40, 7.31)	2.11 (0.9, 4.97)
Any NSAID (Yes)	40 (44.9)/49 (55.1)	4.12 (2.18, 7.79); *p* < .0001	91 (59.1)/63 (40.9)	7.57 (4.43, 12.93); *p* < .0001		
Non-aspirin NSAID (No)	74 (24.3)/230 (75.7)	1	141 (25.0)/422 (75.0)	1.04 (0.71, 1.53); *p* = .8433	3.11 (−0.82, 7.05)	2.03 (0.81, 5.08)
Non-aspirin NSAID (Yes)	34 (45.3)/41 (54.7)	3.99 (2.06, 7.75); *p* < .0001	77 (58.3)/55 (41.7)	7.15 (4.10, 12.46); *p* < .0001		
Aspirin intake (No)	92 (28.8)/227 (71.2)	1.0	170 (29.4)/409 (70.6)	1.09 (0.76–1.54); *p* = .6474	6.04 (−1.89, 13.98)	7.72 (0.38–158.17)
Aspirin intake (Yes)	6 (40.0)/9 (60.0)	1.81 (0.45–7.37); *p* = .4045	18 (69.2)/8 (30.8)	7.94 (2.99–21.13); *p* < .0001		
rs6664:C > T						
Any NSAID (No)	75 (24.4)/233 (75.6)	1	120 (22.9)/403 (77.1)	1.13 (0.76, 1.68); *p* = .5438	−1.45 (−6.05, 3.15)	0.78 (0.37, 1.65)
Any NSAID (Yes)	53 (55.8)/42 (44.2)	7.47 (4.02, 13.88); *p* < .0001	78 (52.7)/70 (47.3)	6.15 (3.60, 10.50); *p* < .0001		
Non-aspirin NSAID (No)	83 (25.6)/241 (74.4)	1	132 (24.3)/411 (75.7)	1.16 (0.79, 1.70); *p* = .4486	−3.37 (−8.77, 2.03)	0.55 (0.24, 1.25)
Non-aspirin NSAID (Yes)	45 (57.0)/34 (43.0)	8.38 (4.33, 16.18); *p* < .0001	66 (51.6)/62 (48.4)	5.16 (3.00, 8.90); *p* < .0001		
Aspirin intake (No)	104 (31.0)/231 (69.0)	1.0	158 (28.1)/405 (71.9)	0.95 (0.67–1.34); *p* = .7793	5.55 (−2.60, 13.70)	5.74 (0.49–67.83)
Aspirin intake (Yes)	8 (47.1)/9 (52.9)	2.22 (0.69–7.17); *p* = .1829	16 (66.7)/8 (33.3)	7.72 (2.75–21.68); *p* = .0001		
rs2283458:A > G						
Any NSAID (No)	107 (26.7)/294 (73.3)	1	88 (20.5)/342 (79.5)	0.69 (0.47, 1.01); *p* = .0532	0.56 (−2.62, 3.73)	1.15 (0.50, 2.64)
Any NSAID (Yes)	62 (53.4)/54 (46.6)	4.92 (2.83, 8.58); *p* < .0001	69 (54.3)/58 (45.7)	5.17 (3.07, 8.70); *p* < .0001		
Non-aspirin NSAID (No)	117 (28.1)/300 (71.9)	1	98 (21.8)/352 (78.2)	0.71 (0.49, 1.03); *p* = .068	0.77 (−2.41, 3.96)	1.24 (0.5, 3.09)
Non-aspirin NSAID (Yes)	52 (52.0)/48 (48.0)	4.46 (2.51, 7.93); *p* < .0001	59 (55.1)/48 (44.9)	4.95 (2.86, 8.57); *p* < .0001		
Aspirin intake (No)	134 (31.7)/289 (68.3)	1.0	128 (26.9)/347 (73.1)	0.78 (0.56–1.08); *p* = .1369	0.02 (−6.07, 6.10)	1.01 (0.14–7.55)
Aspirin intake (Yes)	13 (65.0)/7 (35.0)	4.24 (1.38–13.06); *p* = .0118	11 (52.4)/10 (47.6)	4.03 (1.46–11.15); *p* = .0072		
rs1060463:C > G/C > T						
Any NSAID (No)	98 (22.5)/337 (77.5)	1	97 (24.5)/299 (75.5)	1.36 (0.93, 1.99); *p* = .1143	0.73 (−3.88, 5.33)	1.12 (0.55, 2.29)
Any NSAID (Yes)	67 (52.3)/61 (47.7)	6.67 (3.94, 11.28); *p* < .0001	64 (55.7)/51 (44.3)	7.75 (4.41, 13.62); *p* < .0001		
Non-aspirin NSAID (No)	103 (22.9)/346 (77.1)	1	112 (26.8)/306 (73.2)	1.42 (0.98, 2.05); *p* = .0603	0.019 (−4.65, 4.69)	1 (0.46, 2.19)
Non-aspirin NSAID (Yes)	62 (54.4)/52 (45.6)	6.53 (3.81, 11.19); *p* < .0001	49 (52.7)/44 (47.3)	6.97 (3.81, 12.74); *p* < .0001		
Aspirin intake (No)	138 (28.8)/342 (71.3)	1.0	124 (29.7)/294 (70.3)	1.28 (0.91–1.79); *p* = .1542	2.07 (−5.66, 9.79)	1.62 (0.27–9.83)
Aspirin intake (Yes)	8 (50.0)/8 (50.0)	4.05 (1.26–12.98); *p* = .0186	16 (64.0)/9 (36.0)	6.39 (2.35–17.38); *p* = .0003		
rs1695:A > G						
Any NSAID (No)	94 (24.5)/289 (75.5)	1	101 (22.5)/347 (77.5)	0.82 (0.56, 1.20); *p* = .3084	−0.11 (−3.67, 3.46)	0.98 (0.44, 2.18)
Any NSAID (Yes)	63 (59.4)/43 (40.6)	5.70 (3.27, 9.92); *p* < .0001	68 (49.6)/69 (50.4)	5.41 (3.19, 9.18); *p* < .0001		
Non-aspirin NSAID (No)	101 (25.4)/297 (74.6)	1	114 (24.3)/355 (75.7)	0.90 (0.62, 1.30); *p* = .5776	−1.24 (−5.08, 2.60)	0.75 (0.32, 1.77)
Non-aspirin NSAID (Yes)	56 (61.5)/35 (38.5)	6.08 (3.40, 10.88); *p* < .0001	55 (47.4)/61 (52.6)	4.74 (2.72, 8.27); *p* < .0001		
Aspirin intake (No)	133 (31.4)/290 (68.6)	1.0	129 (27.2)/346 (72.8)	0.83 (0.59–1.17); *p* = .2858	1.14 (−5.04, 7.31)	1.44 (0.18–11.37)
Aspirin intake (Yes)	9 (56.3)/7 (43.8)	3.73 (1.14–12.21); *p* = .0295	15 (60.0)/10 (40.0)	4.70 (1.76–12.52); *p* = .0020		
rs3756067:G > A						
Any NSAID (No)	79 (23.0)/265 (77.0)	1	114 (23.8)/366 (76.3)	0.93 (0.63, 1.38); *p* = .7199	−0.03 (−3.53, 3.46)	0.99 (0.45, 2.18)
Any NSAID (Yes)	57 (50.9)/55 (49.1)	5.52 (3.15, 9.68); *p* < .0001	68 (54.4)/57 (45.6)	5.42 (3.10, 9.46); *p* < .0001		
Non-aspirin NSAID (No)	88 (24.3)/274 (75.7)	1	124 (24.9)/373 (75.1)	0.87 (0.60, 1.27); *p* = .483	0.22 (−3.11, 3.56)	1.06 (0.44, 2.57)
Non-aspirin NSAID (Yes)	48 (51.1)/46 (48.9)	4.79 (2.66, 8.64); *p* < .0001	58 (53.7)/50 (46.3)	4.89 (2.77, 8.64); *p* < .0001		
Aspirin intake (No)	105 (28.2)/267 (71.8)	1.0	150 (29.2)/364 (70.8)	0.94 (0.67–1.32); *p* = .7272	0.08 (−6.60, 6.76)	1.02 (0.15–7.02)
Aspirin intake (Yes)	13 (56.5)/10 (43.5)	4.48 (1.62–12.35); *p* = .0038	10 (58.8)/7 (41.2)	4.50 (1.41–14.40); *p* = .0113		
rs3765070:A > G/A > T						
Any NSAID (No)	99 (22.7)/337 (77.3)	1	96 (24.3)/299 (75.7)	1.32 (0.90, 1.93); *p* = .1534	0.99 (−3.57, 5.54)	1.17 (0.57, 2.40)
Any NSAID (Yes)	66 (52.0)/61 (48.0)	6.46 (3.82, 10.93); *p* < .0001	65 (56.0)/51 (44.0)	7.77 (4.43, 13.61); *p* < .0001		
Non-aspirin NSAID (No)	104 (23.1)/346 (76.9)	1	111 (26.6)/306 (73.4)	1.38 (0.96, 2.00); *p* = .0822	0.31 (−4.32, 4.94)	1.05 (0.48, 2.30)
Non-aspirin NSAID (Yes)	61 (54.0)/52 (46.0)	6.33 (3.69, 10.86); *p* < .0001	50 (53.2)/44 (46.8)	7.02 (3.85, 12.80); *p* < .0001		
Aspirin intake (No)	138 (28.8)/342 (71.3)	1.0	124 (29.7)/294 (70.3)	1.26 (0.9–1.76); *p* = .1772	2.07 (−5.60, 9.75)	1.63 (0.27–9.96)
Aspirin intake (Yes)	8 (50.0)/8 (50.0)	4.02 (1.26–12.88); *p* = .0191	16 (64.0)/9 (36.0)	6.35 (2.34–17.27); *p* = .0003		
rs3869579:G > A/G > C						
Any NSAID (No)	89 (23.1)/296 (76.9)	1	106 (23.8)/340 (76.2)	1.14 (0.78, 1.67); *p* = .5035	−0.33 (−4.53, 3.87)	0.94 (0.45, 1.95)
Any NSAID (Yes)	58 (53.7)/50 (46.3)	6.68 (3.78, 11.79); *p* < .0001	73 (54.1)/62 (45.9)	6.49 (3.82, 11.03); *p* < .0001		
Non-aspirin NSAID (No)	99 (24.6)/303 (75.4)	1	116 (24.9)/349 (75.1)	1.15 (0.80, 1.66); *p* = .4474	−0.63 (−4.86, 3.59)	0.89 (0.4, 1.99)
Non-aspirin NSAID (Yes)	48 (52.7)/43 (47.3)	6.35 (3.50, 11.50); *p* < .0001	63 (54.3)/53 (45.7)	5.87 (3.38, 10.19); *p* < .0001		
Aspirin intake (No)	116 (28.4)/293 (71.6)	1.0	146 (29.9)/343 (70.1)	1.09 (0.78–1.52); *p* = .6258	0.34 (−6.81, 7.50)	1.09 (0.18–6.72)
Aspirin intake (Yes)	10 (58.8)/7 (41.2)	4.69 (1.53–14.36); *p* = .0068	14 (58.3)/10 (41.7)	5.12 (1.85–14.20); *p* = .0017		
rs4715332:C > A						
Any NSAID (No)	98 (23.8)/314 (76.2)	1	97 (23.2)/322 (76.8)	0.88 (0.60, 1.28); *p* = .507	2.34 (−1.49, 6.16)	1.64 (0.74, 3.65)
Any NSAID (Yes)	61 (50.4)/60 (49.6)	4.76 (2.76, 8.22); *p* < .0001	70 (57.9)/51 (42.1)	6.98 (4.09, 11.92); *p* < .0001		
Non-aspirin NSAID (No)	110 (25.3)/324 (74.7)	1	105 (24.3)/327 (75.7)	0.85 (0.59, 1.23); *p* = .3941	2.25 (−1.36, 5.86)	1.75
Non-aspirin NSAID (Yes)	49 (49.5)/50 (50.5)	4.15 (2.31, 7.43); *p* < .0001	62 (57.4)/46 (42.6)	6.25 (3.62, 10.77); *p* < .0001		(0.71, 4.32)
Aspirin intake (No)	125 (28.8)/318 (71.8)	1.0	137 (30.1)/318 (69.9)	0.98 (0.70–1.38); *p* = .9270	1.99 (−6.68, 10.66)	1.61 (0.24–10.9)
Aspirin intake (Yes)	14 (58.3)/10 (41.7)	4.27 (1.62–11.24); *p* = .0033	10 (62.5)/6 (37.5)	6.24 (1.76–22.12); *p* = .0046		
rs4715354:G > A						
Any NSAID (No)	99 (24.7)/302 (75.3)	1	95 (22.1)/334 (77.9)	0.95 (0.65, 1.40); *p* = .8103	1.53 (−2.25, 5.32)	1.37 (0.63, 2.96)
Any NSAID (Yes)	61 (50.4)/60 (49.6)	5.24 (3.03, 9.08); *p* < .0001	70 (57.4)/52 (42.6)	6.73 (3.95, 11.46); *p* < .0001		
Non-aspirin NSAID (No)	108 (25.7)/313 (74.3)	1	106 (23.8)/339 (76.2)	0.95 (0.66, 1.37); *p* = .7886	1.16 (−2.54, 4.85)	1.30 (0.56, 3.04)
Non-aspirin NSAID (Yes)	52 (51.5)/49 (48.5)	4.90 (2.73, 8.79); *p* < .0001	59 (55.7)/47 (44.3)	6.01 (3.48, 10.36); *p* < .0001		
Aspirin intake (No)	127 (29.4)/305 (70.6)	1.0	135 (29.0)/331 (71.0)	1.02 (0.73–1.42); *p* = .9198	0.88 (−6.37, 8.13)	1.26 (0.20–8.05)
Aspirin intake (Yes)	14 (58.3)/10 (41.7)	4.39 (1.61–11.97); *p* = .0039	10 (58.8)/7 (41.2)	5.28 (1.68–16.61); *p* = .0044		
rs1799931:G > A						
Any NSAID (No)	186 (23.6)/601 (76.4)	1	9 (20.5)/35 (79.5)	0.98 (0.42, 2.28); *p* = .9634	−2.49 (−8.06, 3.07)	0.53 (0.08, 3.4)
Any NSAID (Yes)	123 (53.7)/106 (46.3)	6.30 (4.20, 9.46); *p* < .0001	8 (57.1)/6 (42.9)	3.79 (0.98, 14.63)		
Non-aspirin NSAID (No)	203 (24.8)/615 (75.2)	1	12 (24.5)/37 (75.5)	0.99 (0.45, 2.21); *p* = .9854	−2.07 (−8.31, 4.17)	0.56 (0.06, 5.18)
Non-aspirin NSAID (Yes)	106 (53.5)/92 (46.5)	5.74 (3.77, 8.75); *p* < .0001	5 (55.6)/4 (44.4)	3.67 (0.74, 18.11); *p* = .1107		
Aspirin intake (No)	280 (28.9)/690 (71.1)	1	14 (26.4)/39 (73.6)	0.91 (0.43, 1.94); *p* = .8103	−1.63 (−10.30, 7.03)	0.57 (0.01, 24.24)
Aspirin intake (Yes)	29 (63.0)/17 (37.0)	4.87 (2.20, 10.81); *p* = .0001	3 (60.0)/2 (40.0)	3.15 (0.26, 37.77); *p* = .3644		
rs28399433:A > C						
Any NSAID (No)	172 (23.4)/563 (56.6)	1	22 (24.7)/67 (75.3)	0.93 (0.50, 1.72); *p* = .8125	0.23 (−5.51, 5.96)	1.05 (0.34, 3.2)
Any NSAID (Yes)	116 (54.0)/99 (46.0)	6.02 (3.96, 9.15); *p* < .0001	14 (51.9)/13 (48.1)	6.18 (2.50, 15.23); *p* = .0001		
Non-aspirin NSAID (No)	190 (24.8)/577 (75.2)	1	24 (25.8)/69 (74.2)	1.01 (0.56, 1.81); *p* = .9841	−0.86 (−5.92, 4.19)	0.82 (0.23, 2.92)
Non-aspirin NSAID (Yes)	98 (53.6)/85 (46.4)	5.71 (3.69, 8.83); *p* < .0001	12 (52.2)/11 (47.8)	4.85 (1.84, 12.81); *p* = .0014		
Aspirin intake (No)	258 (28.5)/646 (71.5)	1	34 (30.6)/77 (69.4)	0.97 (0.57, 1.64); *p* = .9016	0.025 (−10.43, 10.48)	1.01 (0.06, 17.32)
Aspirin intake (Yes)	30 (65.2)/16 (34.8)	4.69 (2.09, 10.50); *p* = .0002	3 (60.0)/2 (40.0)	4.68 (0.57, 38.56); *p* = .1514		
rs4536:C > T						
Any NSAID (No)	191 (23.6)/618 (76.4)	1	4 (19.0)/17 (81.0)	0.32 (0.06, 1.66); *p* = .1743	−2.80 (−8.08, 2.47)	0.37 (0.02, 7.43)
Any NSAID (Yes)	127 (54.3)/107 (45.7)	6.10 (4.08, 9.12); *p* < .0001	3 (37.5)/5 (62.5)	2.61 (0.41, 16.49); *p* = .3070		
Non-aspirin NSAID (No)	210 (24.9)/633 (75.1)	1	5 (21.7)/18 (78.3)	0.29 (0.06, 1.53); *p* = .1450	−2.38 (−7.41, 2.66)	0.39 (0.02, 8.2)
Non-aspirin NSAID (Yes)	108 (54.0)/92 (46.0)	5.60 (3.69, 8.52); *p* < .0001	2 (33.3)/4 (66.7)	2.52 (0.41, 15.62); *p* = .3213		
Aspirin intake (No)	287 (28.9)/707 (71.1)	1	6 (22.2)/21 (77.8)	0.37 (0.11, 1.27); *p* = .1139	Not applicable	Not applicable
Aspirin intake (Yes)	31 (63.3)/18 (36.7)	4.63 (2.16, 9.91); *p* = .0001	1 (50.0)/1 (50.0)	Not applicable		
rs58285195:T > C						
Any NSAID (No)	173 (22.7)/588 (77.3)	1	22 (31.4)/48 (68.6)	1.57 (0.84, 2.92); *p* = .1553	7.68 (−12.96, 28.31)	2.35 (0.48, 11.4)
Any NSAID (Yes)	122 (53.0)/108 (47.0)	6.10 (4.05, 9.18); *p* < .0001	9 (69.2)/4 (30.8)	14.35 (3.38, 60.98); *p* = .0003		
Non-aspirin NSAID (No)	193 (24.2)/604 (75.8)	1	22 (31.4)/48 (68.6)	5.50 (3.60, 8.42); *p* < .0001	7.23 (−11.67, 26.14)	2.46 (0.5, 12.11)
Non-aspirin NSAID (Yes)	102 (52.6)/92 (47.4)	5.50 (3.60, 8.42); *p* = .2383	9 (69.2)/4 (30.8)	13.18 (3.12, 55.72); *p* = .0005		
Aspirin intake (No)	264 (28.0)/678 (72.0)	1	30 (37.0)/51 (63.0)	1.70 (0.96, 3.02); *p* = .0679	Not applicable	Not applicable
Aspirin intake (Yes)	31 (63.3)/18 (36.7)	5.36 (2.45, 11.73); *p* < .0001	1 (50.0)/1 (50.0)	Not applicable		

SNP: single nucleotide polymorphism.

**^†^**Odds Ratio adjusted for: period of patients’ recruitment, previous history of arthrosis, infection with *Helicobacter pylori*, gastrointestinal disorders (ulcer and bleeding), exposure to inhibitors of the proton pump, exposure to antiaggregant, exposure to anticoagulants, and the interview variables (the number and the reliability of the interview).

#### 3.3.2. Genotypes associated with moderate excess of risk of UGIH

An increased odds of UGIH was observed from any NSAID, non-aspirin NSAIDs, or aspirin intake by both the carriers of the genetic variants (rs4715332:C>A and rs4715354:G>A) or their corresponding wild-type genotype (Table 4). However, carriers of the genetic variation were at higher odds of UGIH than carriers of the wild-type genotype. A moderate non-statistically significant excess risk was observed for the presence of these genetic variants: rs4715332:C>A [any NSAID (*S* = 1.64; RERI = 2.34), non-aspirin NSAIDs (*S* = 1.75; RERI = 2.25) and aspirin (*S* = 1.61; RERI = 1.99)] and rs4715354:G>A [any NSAID (*S* = 1.37; RERI = 1.53), non-aspirin NSAIDs (*S* = 1.30; RERI = 1.16) and aspirin (*S* = 1.26; RERI = 0.88)] (Table 4).

Similar observations were observed for aspirin users carrying rs6664:C>T. Aspirin users carrying this genetic variant had substantially higher odds of UGIH in comparison with patients carrying the wild-type genotype [OR_wild-type_: 2.22 (95%CI: 0.69–7.17) *vs.* OR_genetic-variation_: 7.72 (95%CI: 2.75–21.68)]. Nonetheless the number of aspirin users in this subgroup was limited which yielded a non-statistically significant measure of interaction [*S* = 5.74 (95%CI: 0.49–67.83); RERI = 5.55 (95%CI: −2.60–13.70)]

#### 3.3.3. Genotypes not associated with modification of the risk of UGIH

Carriers of the wild-type genotypes and carriers of the following genetic variants are associated with a similar magnitude of risk of UGIH: rs2283458:A>G, rs1060463: C>G/C>T, rs1695:A>G, rs3756067:G>A, rs3765070:A>G/A>T, rs3869579:G>A/G>C, rs28399433:A>C (Table 4).

The risk associated with rs36079186 could not be determined because it was monovariant (the same genotype was observed in all the study population). Inconclusive results were also obtained for rs4536:C>T and rs58285195:T>C due to the limited number of cases and controls who are on NSAIDs treatment and carriers for these genetic variations.

## 4. Discussion

To the best of our knowledge, this is the first study that finds a statistically significant additive synergism interaction between certain genetic polymorphisms in genes involved in drug metabolism (rs2180314:C>G and rs4809957:A>G) and NSAIDs on UGIH. Our results indicate that the joint effect of these SNPs with NSAIDs is more than three times higher [rs2180314:C>G (*S* = 3.30; 95%CI: 1.24, 8.80), rs4809957:A>G (*S* = 2.11; 95%CI: 0.90, 4.97)] than the sum of their individual effects. Since (1) UGIH contributes to high mortality and morbidity rates [28], (2) NSAIDs are among the most commonly used medicines worldwide [4], (3) a large fraction of the European population carries the genetic variants rs2180314:C>G (58%) and rs4809957:A>G (21%) [53], and (4) genotyping is a low-cost test, our findings could enable identifying better the individuals at risk and those who are not at risk of UGIH from NSAID exposure.

The interindividual variations in therapeutic responses to NSAIDs were associated earlier with several demographic and clinical factors. Variations in patients’ response to aspirin NSAIDs were also suggested to be related to the metabolic rate of this drug and the excretion of aspirin metabolites [54]. Patients are classified as fast and slow acetylators, and these metabolic differences were associated with genetic factors [55].

We are not aware of any previous study that evaluated the interaction between the polymorphisms tested in the present work and NSAID (non-aspirin and aspirin) on UGIH. Consequently, it was not possible to compare our findings to that of other studies. Few studies examined the associations between the genetic variants assessed in the current study and other gastrointestinal disorders (ulcers or small bowel bleeding). Shiotani and colleagues suggested a possible relation between the genetic variants rs2180314:G>C, rs4809957:A>G, rs1060463:C>G/C>T, and rs1695:A>G and the risk of small bowel bleeding in Japanese patients on aspirin therapy, however, no significant association was demonstrated [25]. Another study in Japan also reported a significant association of rs6664:C>T genotype with the risk of ulcer bleeding in aspirin users [26]. Shiotani and colleagues also suggested an association between rs3765070:A>G/A>T, rs4715354:G>A, rs2283458:A>G, and rs3756067:G>A and small bowel bleeding in a genome-wide analysis in the Japanese population; nevertheless, the association disappeared upon the validation of those SNPs [25].

The explanation of the increased odds of UGIH in NSAID consumers who are carriers of the genetic variations rs2180314:C>G or rs4809957:A>G is limited. However, we hypothesize that this excess in risk could be a consequence of an altered function of the corresponding genes due to polymorphisms. rs2180314:C>G belongs to the glutathione S-transferase (GSTA) gene family which plays an important role in the detoxification of electrophilic compounds, including therapeutic drugs, and protects the cells against damage. GSTA genes are highly polymorphic, and their genetic variation may alter the toxicity and efficacy of some drugs [56,57]. rs4809957:A>G belongs to gene members of the P450 family. Enzymes encoded by P450 are monooxygenases that catalyze many drug metabolism reactions [58].

In general, testing a large number of SNPs increases type 1 error, and therefore increments the chance of obtaining false-positive conclusions [59]. Nevertheless, in this study, we attempted to minimize type 1 error by performing a pre-hoc SNP selection whereby we chose specifically those SNPs that belong to genes involved in drug metabolism, and which were suggested to be associated with gastrointestinal disorders [25,26]. Furthermore, we analyzed each SNP in an independent model and reported all the implemented analyses. Our strategy of SNP selection and data reporting exempts from adjusting for multiple testing and leads to fewer errors of interpretation [60,61]. Another strength of this study is the control for all possible biases. The memory bias was reduced by showing prompt cards of the most frequent NSAID commercial boxes to the patients during the interview and by reviewing the medical records. The exclusion of non-white patients also allowed to prevent bias due to racial differences between populations. Moreover, performing the study in biologically unrelated patients, exclusively, avoided the over-representation of the bias of genotype within families. Finally, the measures of effect reported in this study were all adjusted to baseline risk factors that were known to increase the risk of gastrointestinal bleeding.

The main limitation of our study was the sample size. In fact, upon stratification by genotype and types of NSAID, a limited number of observations were left in the subgroups mainly for aspirin, which consequently decreased the statistical power for the associations of many SNPs. Low statistical power is a frequent limitation in candidate gene studies [62,63]. Another consequence imposed by the modest sample size is the curse of dimensionality; i.e. the number of observations was very small when various SNPs were combined [64]. Therefore, it was not feasible to analyze the combined effect of different genetic variations. In addition, it was not possible to undertake a dose-response analysis. Therefore, we believe that further studies with a larger sample size are needed to confirm these results before their implantation in clinical settings. Another limitation of our study is the potential presence of false *H. pylori* test results. Though we intended to minimize the false positive rate by inquiring about treatment for *H. pylori* infection in the past, we cannot rule out the possibility of having false negative results since the rate of eradication of *H. pylori* from a single treatment varies between 60% and 90% [65].

In conclusion, this study revealed that genetic variations might alter the pharmacological and clinical response to NSAIDs. The risk of UGIH in NSAID users with the wild type genotypes of rs2180314 and rs4809957 is significantly lower than that in those users who carry the genetic variants. If our results were confirmed by future studies, they would suggest that simple genetic profiling, a low-cost test, can be used to support a clinical decision towards personalized NSAIDs prescription. These findings are of important clinical relevance since NSAIDs (non-aspirin and aspirin) are among the most frequently prescribed drugs due to their wide spectrum of benefits and cost-efficiency. The medical community needs to carefully weigh the benefits and risks of NSAIDs for each patient and take measures that maximize the benefits of these drugs.

## Data Availability

The data that support the findings of this study are available in [FigShare] at [10.6084/m9.figshare.11822223].
